# Pilot-Scale Electrospinning of PLA Using Biobased Dyes as Multifunctional Additives

**DOI:** 10.3390/polym14152989

**Published:** 2022-07-23

**Authors:** Naveen Kumar Balakrishnan, Maike-Elisa Ostheller, Niccolo Aldeghi, Christian Schmitz, Robert Groten, Gunnar Seide

**Affiliations:** 1 Aachen-Maastricht Institute for Biobased Materials (AMIBM), Maastricht University, Brightlands Chemelot Campus, Urmonderbaan 22, 6167 RD Geleen, The Netherlands; m.ostheller@maastrichtuniversity.nl (M.-E.O.); niccolo.aldeghi@noosafiber.com (N.A.); christian.schmitz@maastrichtuniversity.nl (C.S.); gunnar.seide@maastrichtuniversity.nl (G.S.); 2 Department of Textile and Clothing Technology, Niederrhein University of Applied Sciences, Campus Moenchengladbach, Webschulstrasse 31, 41065 Moenchengladbach, Germany; robert.groten@hs-niederrhein.de

**Keywords:** melt electrospinning, PLA, antibacterial, biobased dyes, alizarin, curcumin, quercetin, environmental sustainability

## Abstract

Fibers with diameters in the lower micrometer range have unique properties suitable for applications in the textile and biomedical industries. Such fibers are usually produced by solution electrospinning, but this process is environmentally harmful because it requires the use of toxic solvents. Melt electrospinning is a sustainable alternative but the high viscosity and low electrical conductivity of molten polymers produce thicker fibers. Here, we used multifunctional biobased dyes as additives to improve the spinnability of polylactic acid (PLA), improving the spinnability by reducing the electrical resistance of the melt, and incorporating antibacterial activity against Staphylococcus aureus. Spinning trials using our 600-nozzle pilot-scale melt-electrospinning device showed that the addition of dyes produced narrower fibers in the resulting fiber web, with a minimum diameter of ~9 µm for the fiber containing 3% (*w*/*w*) of curcumin. The reduction in diameter was low at lower throughputs but more significant at higher throughputs, where the diameter reduced from 46 µm to approximately 23 µm. Although all three dyes showed antibacterial activity, only the PLA melt containing 5% (*w*/*w*) curcumin retained this property in the fiber web. Our results provide the basis for the development of environmentally friendly melt-electrospinning processes for the pilot-scale manufacturing of microfibers.

## 1. Introduction

Microfibers, sub-microfibers, and nanofibers have a large surface area to volume ratio, making them ideal for applications as textiles (e.g., material for filters), biomedical devices (e.g., wound dressings, tissue engineering constructs, and drug delivery vehicles) [1], and battery components for fuel and solar cells [1,2,3,4,5,6]. The unique properties of such fibers include better flexibility, waterproofing, and breathability compared to regular fibers [7,8,9]. Their production methods include melt blowing, electro-blowing, and centrifugal spinning, but the most widely used one is electrospinning [10].

Electrospinning involves the application of an external electrostatic field to a polymer liquid as they emerge from a nozzle [11]. The electrostatic repulsion caused by this charge is strong enough to overcome the surface tension of a droplet, and once a certain threshold is exceeded, it results in the formation of a Taylor cone [12,13]. If the liquid is viscous and cohesive, it does not break up into droplets (the principle of electro-spraying) but forms an electrically charged laminar jet, which elongates due to electrostatic repulsion. In solution electrospinning, the fiber is formed as the solvent evaporates; in melt electrospinning, it is formed as the polymer solidifies.

The diameter of the fibers is mainly dependent on the viscosity and electrical conductivity of the polymer solution or melt. Solution electrospinning is used most frequently because polymer solutions are less viscous and more conductive than molten polymers, leading to more interaction with the electric field and enhanced whipping behavior of the polymer jet, producing finer fibers. However, for polymers such as polylactic acid, commonly used solvents include chloroform, dichloromethane, and N, N-dimethylformamide. Furthermore, solvents make up the bulk of the raw material. The polymer content is therefore low (reducing productivity), and this is exacerbated by frequent nozzle blockages caused by solvent evaporation [11,14]. Furthermore, even trace amounts of solvents present in the fibers create problems during usage, especially in the medical field, and therefore extensive removal of solvent is necessary. Therefore, researchers are shifting towards the more sustainable melt-electrospinning approach. However, the higher viscosity and lower electrical conductivity of molten polymers make the production of sub-microfibers more challenging. This can be addressed in part by modifying the electrospinning equipment [1,15,16]. Examples include the use of multi-temperature control [15] and spot laser melt electrospinning to produce fibers in the sub-micrometer range [16]. Another approach is the use of additives that alter the properties of the molten polymer, such as salts to improve the conductivity of polymers [17] or plasticizers to reduce the viscosity of polymers [10]. We have previously used dyes and pigments as additives to increase the conductivity of molten PLA during electrospinning and successfully reduced the diameter of the fibers [18,19]. However, the addition of salt has led to the degradation of PLA in the past.

One of the promising alternatives here is multifunctional colorants. They have been extensively used in the textile field for dyeing, but colorants can also confer other properties, such as conductivity and antibacterial activity. For example, the dye alizarin is used extensively in solar cells and is also researched for other electronic applications [20]. The antibacterial activity of alizarin [21], quercetin [22], and curcumin [23,24] makes them suitable for applications such as food packaging [25] and wound dressings [26]. However, to the best of our knowledge, these additives have been used mainly in solution processing methods or as coatings, with only limited investigation of melt electrospinning. Furthermore, studies on electrospinning also involve single nozzles largely, and pilot-scale melt-electrospinning studies are scarce.

In this study, we investigated the use of the multifunctional biobased dyes alizarin, curcumin, and quercetin as additives to reduce the diameter of PLA fibers produced using a 600-nozzle pilot-scale melt-electrospinning device. We studied the effect of these additives on the thermal and rheological properties of PLA, its degradation, electrical conductivity, and the diameter of the resulting fibers. Furthermore, we investigated the effect of thermal processing on the antibacterial properties of these dyes and the ability of the dyes to confer antibacterial properties on the PLA fibers. Our results provide the basis for the development of environmentally friendly melt-electrospinning processes for the pilot-scale manufacturing of microfibers with diameters in the low micrometer range.

## 2. Materials and Methods

### 2.1. Materials

We used PLA L130 (TotalEnergies|Corbion, Gorinchem, Netherlands), which has an l-PLA content > 99%, a glass transition temperature (*T_g_*) of 60 °C, a melting point (*T_m_*) of 175 °C, and a melt flow index of 24 g/10 min at 210 °C/2.16 kg. The dyes alizarin, curcumin, and quercetin were purchased from Sigma-Aldrich (Zwijndrecht, The Netherlands). All materials were dried at 80 °C overnight before use. The chemical structures and the melting points of the dyes are presented in Table 1.

PLA is typically melt-processed at less than 250 °C. Based on the melting points of the dyes used, we expected curcumin to be a melt during processing and the other two dyes to be solid particles. This could influence the viscosity of the PLA melt. Additionally, the dyes used here also have functional polar groups and cyclic structures with double bonds, and we expected this to lead to improved electrical conductivity.

### 2.2. Methods

#### 2.2.1. Compounding

We prepared the 5% (*w*/*w*) PLA/dye composites using a KETSE 20/40 twin-screw compounder (Brabender, Duisburg, Germany). The compounder featured six heating zones, all of which were set to 200 °C for the compounding trials. The extrudate was cooled in a water bath before granulation.

#### 2.2.2. Pilot-Scale Melt-Electrospinning Equipment

**Figure 1:**
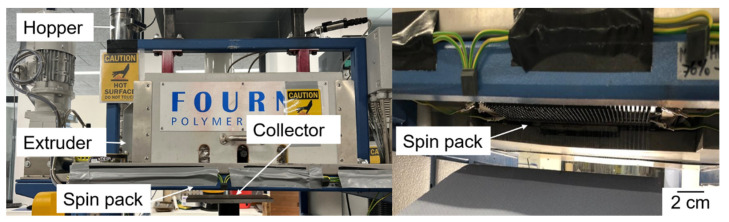
Our 600-nozzle pilot-scale electrospinning machine.

Melt electrospinning was carried out using a pilot-scale machine from Fourne Polymertechnik (Alfter, Germany) with 600 0.3-mm nozzles (Figure 1) to obtain a nonwoven web. We maintained a constant nozzle-to-collector distance of 7 cm and electric field strength of 60 kV. We used three different spin pump speeds (throughputs) of 2, 5, and 10 rpm to investigate the effect of throughput, dye, and the dye weight percentage on the diameter of the fibers. We mixed the 5% (*w*/*w*) PLA/dye composites with pure PLA to make fiber webs containing three different weight percentages of each additive (Table 2) at a spinning temperature of 230 °C.

#### 2.2.3. Characterization of Raw Materials

The rheological properties of PLA were determined using a plate–plate Discovery HR1 hybrid rheometer (TA Instruments, New Castle, DE, USA). The optimal melt-electrospinning temperature for PLA was identified in a time sweep with a 25-mm plate for 60 min at an angular frequency of 10 rad/s, applying a strain amplitude of 5% at 210, 230, and 250 °C.

We determined the thermal stability of the dyes by thermogravimetric analysis (TGA) using a Q500 device (TA Instruments). We tested the dyes by heating from 35 to 230° C and then applied an isothermal run for 60 min to investigate weight loss under nitrogen purge. We also visually inspected the dye color after thermal treatment.

The antibacterial activity of the dyes was tested against Gram-negative *Escherichia coli* ATCC 25922 and Gram-positive *Staphylococcus aureus* ATCC 6538 by assessing the number of colony-forming units. We inoculated five tubes containing 5 mL of lysogeny broth (LB) liquid medium, using 10 µL from the saturated bacterial starting culture as an inoculum before adding 100 µL of each dye at various concentrations (2, 5, 10, 15, and 20 mg/mL) in DMSO. After incubation at 37 °C and 180 rpm for 24 h, we prepared serial dilutions to achieve a bacterial concentration of ~10^5^/mL, and 100-µL aliquots were plated on a tryptic soy agar solid medium. We counted the colonies after incubating the plates at 37 °C for 24 h. We used an untreated culture and a culture containing 100 µL of DMSO as controls.

We investigated physical changes (phase changes/melting) in the dyes within the processing window by differential scanning calorimetry (DSC) on a DSC 214 Polyma device (NETZSCH, Selb, Germany). We tested 5-mg samples by applying a temperature sweep from 25 to 250 °C at a heating rate of 10 °C/min and report the information from the first heating cycle.

#### 2.2.4. Characterization of Composites

Potential interactions between PLA and the dyes were evaluated by Fourier transform infrared spectroscopy (FTIR) using a Perkin Elmer Spotlight 400 FTIR instrument equipped with a diamond crystal in ATR mode. We swept the wavenumber range from 4000 to 600 cm^−1^ at a resolution of 2 cm^−1^ using an average of 64 scans. The background was recorded before composite analysis.

The effect of dyes on the molecular weight of the manufactured master batches was determined by gel permeation chromatography (GPC) using a 1260 Infinity System (Agilent Technologies, Santa Clara, CA, USA). We tested pure PLA and PLA composites containing 5% (*w*/*w*) of each dye, with hexafluor-2-isopropanol (HFIP) containing 0.19% sodium trifluoroacetate as the mobile phase at a flow rate of 0.33 mL/min. Solutions were prepared by dissolving 5 mg of each sample in HFIP for ~2 h and passing them through a 0.2-µm polytetrafluoroethylene filter before injecting them into a modified silica column filled with 7-µm particles (Polymer Standards Service, Mainz, Germany). The relative weight, average molecular weight (Mw), number average molar mass (Mn), and polydispersity index (PDI) were determined using detectors calibrated with poly(methyl methacrylate) standards (1.0 × 10^5^ g/mol).

The effect of dyes and processing on the thermal properties of PLA was tested by DSC, applying a heating and cooling cycle from 25 to 250 °C at a rate of 10 °C/min. We recorded the *T_m_*, *T_g_*, cold crystallization temperature (*T_cc_*), and crystallization temperature (*T_c_*) of PLA and the composites from the first heating and cooling cycle.

We determined the thermal stability of the composites by thermogravimetric analysis (TGA), as mentioned above. Additionally, we also performed a time sweep at 230 °C using the HR1 rheometer on the PLA composites to investigate their thermal stability, as mentioned above. After the isothermal run, we heated the materials up to 500 °C to investigate the effect of the addition of dyes on the 50% weight loss temperature.

#### 2.2.5. Characterization of Melt-Electrospun Fiber Webs

**Figure 2:**
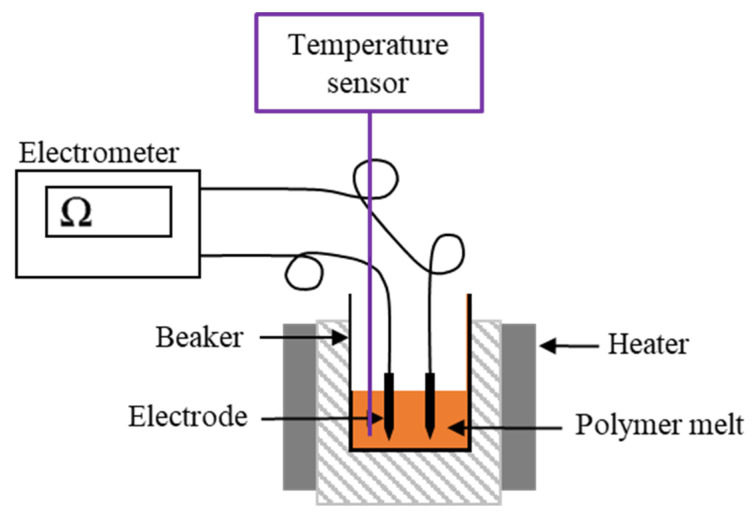
Schematic illustration of our electrometer setup.

We measured the effect of dyes on PLA viscosity using the HR1 rheometer described above. We used a 25-mm plate (gap = 1000 µm) and varied the angular frequency from 10 to 628 rad/s while maintaining the strain amplitude at 5% in a constant environment with a nitrogen atmosphere at 230 °C. The equilibration time was 1 min. To facilitate comparison, we compared the complex viscosity of PLA webs with and without dyes at an angular frequency of 10 rad/s. We recerroorded the electrical resistance of PLA and PLA/dye composite webs at a temperature of 230 °C using a Keithley 617 electrometer (Tektronix, Beaverton, OR, USA), including band heaters to melt the polymer and two electrodes connected to the electrometer in the melt (6 mm apart). We placed a temperature sensor on the polymer melt to measure the melt temperature. We used a 10 MΩ resistor for calibration and applied a potential difference of 10 V between the electrodes (Figure 2).

We measured fiber diameters by reflected light microscopy using a BA310 microscope equipped with Moticam5 + 5.0MP (Motic, Wetzlar, Germany) at 400× magnification. Diameters were measured in 100 images representing different areas of each web and the average value is presented. The webs were also analyzed by DSC in the temperature range 25–250 °C at a heating rate of 10 °C/min. We analyzed all fibers prepared with different throughputs and calculated the crystallinity of the web from the first heating cycle using Equation (1):

(1) Xc (%)=ΔHmΔHm0×100

where *Xc* is the percentage crystallinity, ΔH*m* is the melting enthalpy of the web, and ΔH*m*^0^ is the melting enthalpy of 100% crystalline PLA (93.7 J/g) [28].

We tested the antibacterial properties of the fiber webs against *S. aureus* using the colony counting method discussed above.

## 3. Results and Discussion

### 3.1. Overall Experimental Approach

**Figure 3:**
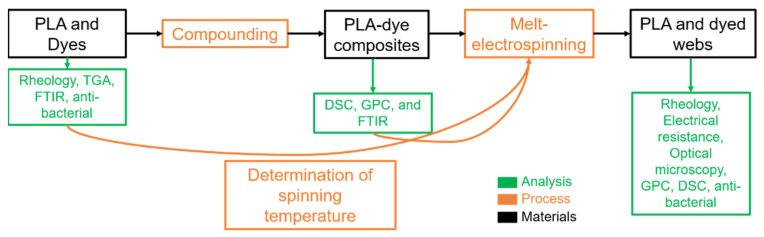
Overview of the experimental approach.

Our experimental approach is summarized in Figure 3. First, we analyzed PLA and the dyes separately to determine the melt-electrospinning temperature. Then, we prepared PLA containing 5% (*w*/*w*) of each dye as composites using a twin-screw compounder and analyzed the effect of the dyes on the thermal properties and thermal stability of PLA. Next, we dry-mixed pure PLA with the PLA dye composites to prepare composites with different weight ratios of each additive (Table 2). Finally, we prepared fibers from the composites using our pilot-scale melt-electrospinning machine. Our primary goal was to reduce the diameter of the fibers by increasing the electrical conductivity and/or reducing the viscosity of the melt. The secondary goal was to incorporate the antibacterial property of the dyes into the melt-electrospun fibers. Other process parameters, such as temperature, electrical field, throughput, and nozzle-to-collector distance, were kept constant so that any change in fiber diameter could be attributed to the dye.

### 3.2. Characterization of Materials

#### 3.2.1. Rheological Analysis of PLA

**Figure 4:**
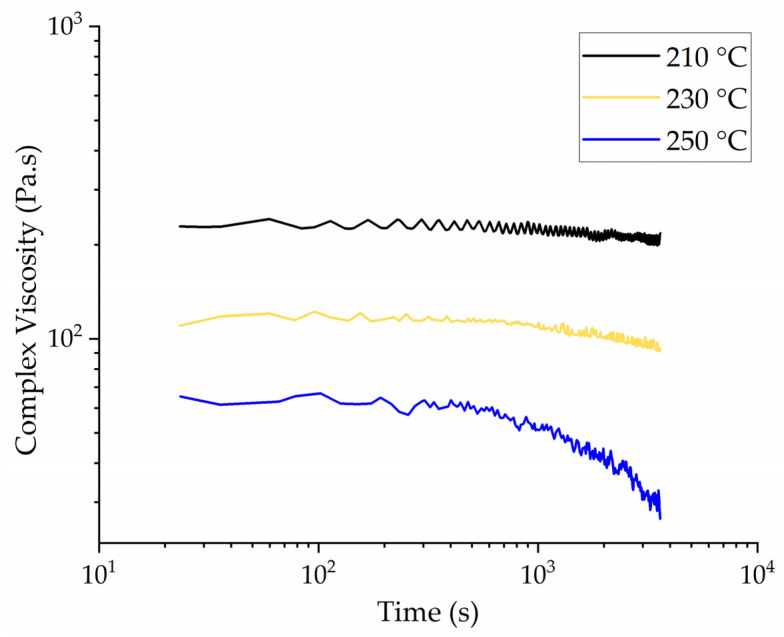
Rheograms representing a time sweep of PLA at different temperatures, showing a loss of complex viscosity at 250 °C.

The rheograms of PLA at 210, 230, and 250 °C are presented in Figure 4. The complex viscosity of PLA was lower at 250 °C than at 210 °C, as expected because the polymer chains become more mobile and therefore the viscosity falls at higher temperatures. The complex viscosity remained constant over time at 210 °C. At 230° C, we observed a slight reduction in the viscosity towards the end of the isothermal run, whereas the reduction was more significant at 250 °C. This suggests that PLA degradation is significant at 250 °C but only marginal at 230 °C. A lower viscosity is preferred for melt electrospinning because whipping behavior increases at lower viscosities. The reduction in complex viscosity was insignificant at 230 °C, so we selected this temperature for the melt-electrospinning experiments.

#### 3.2.2. Thermal Stability of the Dyes

**Figure 5:**
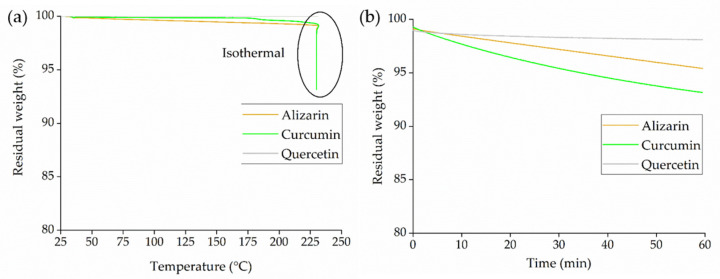
TGA thermogram of dyes during (**a**) the heating ramp to 230 °C and (**b**) the isothermal step at 230 °C for 60 min.

The TGA thermogram of the dyes (Figure 5) indicated negligible weight loss during the heat ramp to 230 °C. Quercetin showed no weight loss even during the isothermal run. However, alizarin and curcumin both lost ~5% during the isothermal run at 230 °C. There was no color change in any of the three dyes following TGA, so we assume that the observed weight loss reflects the loss of water bound in the crystalline region of the dyes. This was not expected to affect the processing of the melt because we dried the dyes prior to the spinning and compounding trials.

#### 3.2.3. Antibacterial Activity of the Dyes

**Figure 6:**
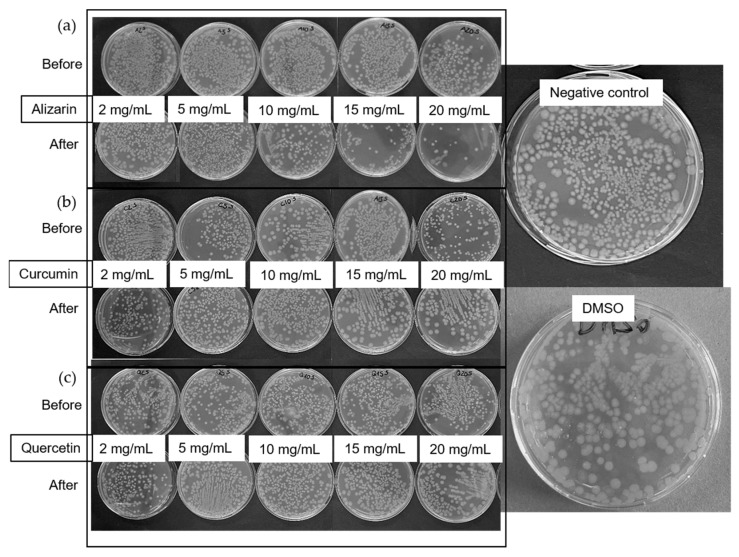
Antibacterial activity testing of (**a**) alizarin, (**b**) curcumin, and (**c**) quercetin against *E. coli* before and after thermal treatment of the dyes at 230 °C.

**Figure 7:**
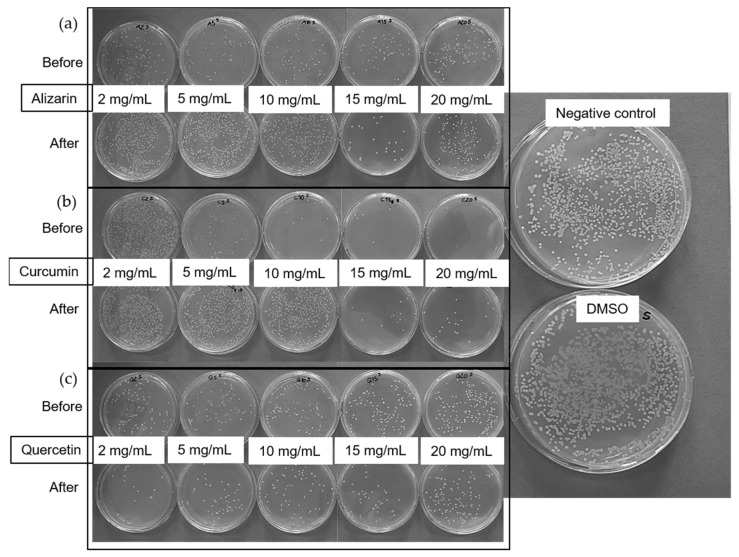
Antibacterial activity testing of (**a**) alizarin, (**b**) curcumin, and (**c**) quercetin against *S. aureus* before and after thermal treatment of the dyes at 230 °C.

The antibacterial activity of the three dyes was tested using the colony counting method against *E. coli* (Figure 6) and *S. aureus* (Figure 7). The negative control and DMSO had no influence on the bacteria, so any reduction in the number of colonies was attributed to the antibacterial activity of the dyes. Alizarin was active against *E. coli* after treatment at 230 °C, but only at concentrations exceeding 10 mg/mL (Figure 6). The other two dyes showed no activity against *E. coli*. One possible explanation for the heat-dependent behavior of alizarin is its ability to undergo keto-enol tautomerization, resulting in a different chemical structure [29]. However, a more plausible reason is the ability of alizarin to form polymorphic crystals [30]. Given that the thermal treatment is close to the melting point of alizarin, a change in crystal structure is likely. Further experiments are needed to determine which feature of alizarin underlies the variable antibacterial activity against *E. coli*.

In contrast to the *E. coli* experiments, all three dyes were active against *S. aureus* (Figure 7). Before thermal treatment, alizarin showed antibacterial activity at the lowest tested concentration of 2 mg/mL. Curcumin was active at concentrations exceeding 5 mg/mL, and quercetin was active at concentrations exceeding 2 mg/mL, in agreement with earlier reports [24]. However, the activity of alizarin and curcumin was diminished by heat treatment, and post-treatment concentrations of more than 15 mg/mL were required to exert an antibacterial effect. Curcumin is also prone to keto-enol tautomerization and it is more active in its keto form [31]. Furthermore, we melted the curcumin and crystallized it again during the thermal treatment, and this could produce a different crystalline form, as discussed above for alizarin. Quercetin appeared to be unaffected by thermal treatment. Given the limited activity of the dyes against *E. coli*, the antibacterial testing of fiber webs was restricted to *S. aureus*.

#### 3.2.4. Thermal Analysis of the Dyes

**Figure 8:**
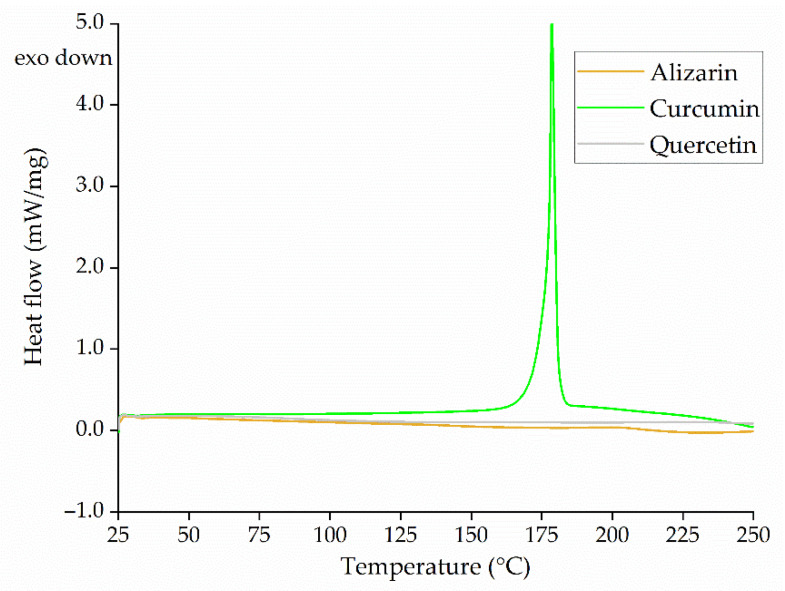
DSC thermogram representing dyes at different temperatures, showing an endothermic peak for curcumin.

The DSC thermogram of the dyes (Figure 8) reveals an endothermic peak at ~175 °C for curcumin, which corresponds to its melting peak. The melting points of alizarin and quercetin fall outside our selected temperature range. Curcumin is a smaller molecule than PLA and its melting point falls below the selected processing temperature of 230 °C, so it could act as a plasticizer, thus reducing melt viscosity. No other thermal transitions were observed for any of the dyes in the selected temperature range, indicating that alizarin and quercetin remained as solid particles in the PLA melt during processing. They could potentially act as anchor points to increase the viscosity of the melt, increasing the viscosity of the PLA compounds.

### 3.3. Characterization of Composites

#### 3.3.1. Analysis of PLA and Its Composites by GPC

**Figure 9:**
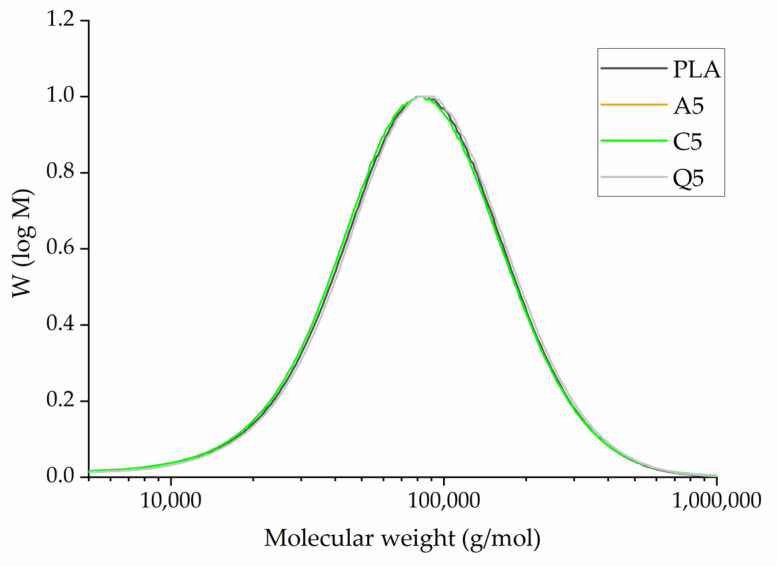
GPC elugram and the Mw, Mn, and PDI of pure PLA and its composites containing 5% (*w*/*w*) of alizarin (A5), curcumin (C5), or quercetin (Q5).

The GPC elugram of PLA and its composites indicates that the average molecular weights (Mw, Mn) of PLA are similar to those of the PLA compounds containing 5% (*w*/*w*) dyes (Figure 9). The values are summarized in Table 3. The highest Mn (~58,700 g/mol) and highest Mw (~108,000 g/mol) were observed for compound Q5. The lowest Mn (~53,000 g/mol) and Mw (~99,000 g/mol) were observed for compound C5. The polydispersity indices (PDIs) were also similar. We concluded that the addition of dyes did not lead to the degradation of PLA during processing.

#### 3.3.2. Analysis of PLA and Its Composites by FTIR Spectroscopy

**Figure 10:**
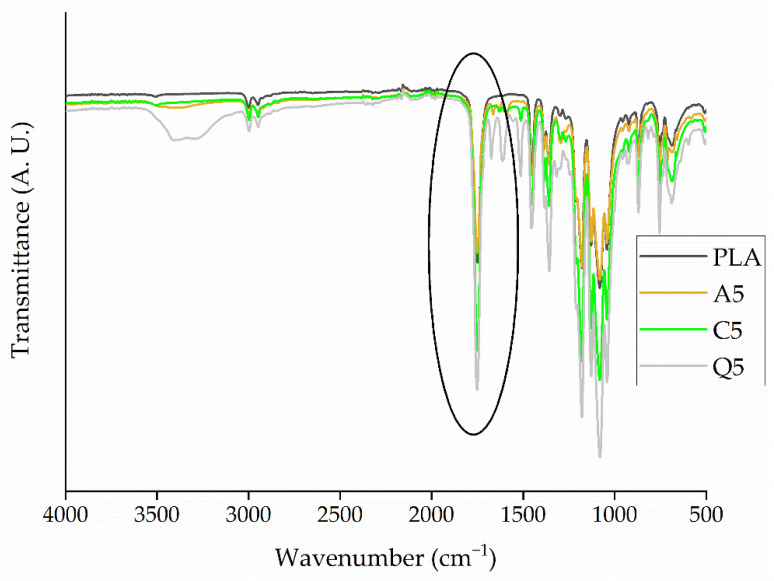
FTIR spectra of pure PLA and its composites containing 5% (*w*/*w*) of alizarin (A5), curcumin (C5), or quercetin (Q5).

The FTIR spectra of PLA and its composites containing 5% (*w*/*w*) of each dye were also very similar (Figure 10). We observed the characteristic C-O bending at 1184 cm^−1^, the -CH_3_ asymmetric and symmetric stretching bands at 1452 cm^−1^ and 1361 cm^−1^, respectively, and the C-O-C stretching band at 1180 cm^−1^. We did not observe a significant change in the positions of these bands in the presence of the dyes [32]. However, the C=O stretching vibration shifted from 1749 cm^−1^ for pure PLA to 1752 cm^−1^ for composite A5 and to 1751 cm^−1^ for composites C5 and Q5. This may indicate hydrogen bonding between the C=O in PLA and the functional groups of the dyes, as observed when PLA was compounded with Kenaf fibers [33]. The presence of hydrogen bonds could increase the melt viscosity by hindering the movement of PLA chains, which we investigated by rheological analysis.

#### 3.3.3. Thermal Stability of PLA and Its Composites

**Figure 11:**
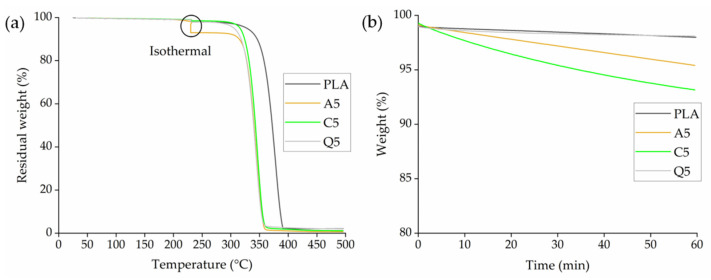
TGA thermogram of PLA and its composites containing 5% (*w*/*w*) of alizarin (A5), curcumin (C5), or quercetin (Q5) during (**a**) the heating ramp from 25 to 230 °C and (**b**) the isothermal step for 60 min at 230 °C.

TGA thermograms of PLA and its composites containing 5% (*w*/*w*) of each dye indicated no loss of weight during the heating ramp up to 230 °C (Figure 11a). Furthermore, PLA and composite Q5 showed negligible weight loss during the isothermal step, whereas composite A5 lost 3% of its weight and composite C5 lost 5% (Figure 11b). We expect this to come from loss of water and these results are similar to the thermal stability analysis of the dyes, and the minimal loss of weight should not affect the processability of PLA. Furthermore, the color of the master batch also remained the same before and after the thermal analysis. It was also observed that PLA has a 50% weight loss temperature of 372 °C and the thermal stability was reduced upon the addition of alizarin and curcumin. The 50% weight loss temperature was reduced to 340 °C for the PLA dye composites. As explained previously in the TGA of dyes, this could be the accelerated degradation occurring because of the release of bound water at higher temperatures. Therefore, it can be concluded that the addition of dyes accelerates the degradation of PLA at higher temperatures when an isothermal step is involved.

**Figure 12:**
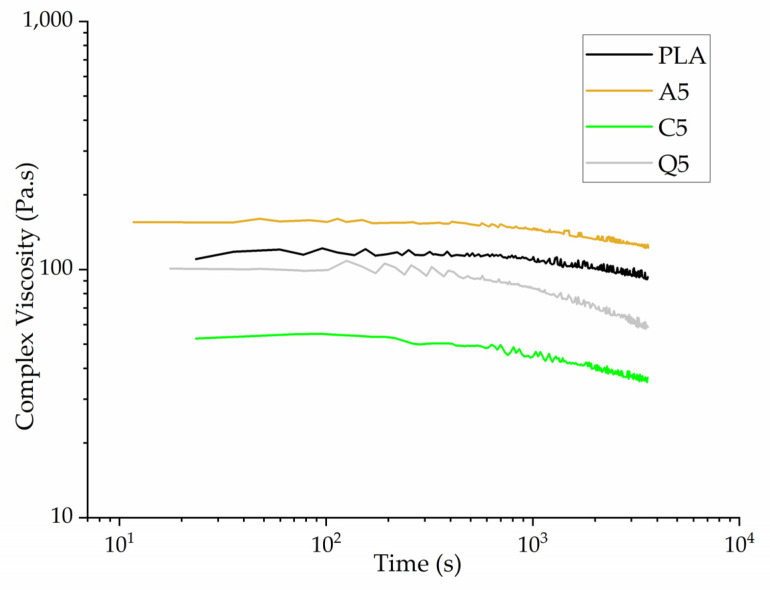
Isothermal rheogram of PLA and its composites containing 5% (*w*/*w*) of alizarin (A5), curcumin (C5), or quercetin (Q5).

The isothermal rheogram of PLA and its composites showed that the complex viscosity of PLA and composite A5 remained mostly constant during isothermal testing at 230 °C, with only a slight degradation toward the end of the isothermal cycle, whereas the complex viscosity of composites Q5 and C5 declined toward the end of the isothermal testing (Figure 12). The complex viscosity of C5 fell from ~50 to ~36 Pa.s, whereas that of Q5 fell from ~100 to ~58 Pa.s. This indicates the slight degradation of PLA in the presence of curcumin or quercetin, but the effect was only marginal and was not expected to affect the melt-electrospinning trials at 230 °C.

#### 3.3.4. Thermal Analysis of PLA and Its Composites

**Figure 13:**
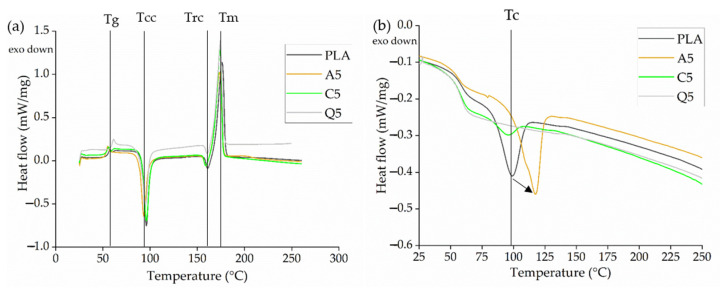
DSC thermograms of PLA and its composites containing 5% (*w*/*w*) of alizarin (A5), curcumin (C5), or quercetin (Q5) during (**a**) the heating cycle and (**b**) the cooling cycle.

The DSC thermograms of PLA and all three composites (Figure 13a) included the glass transition temperature (*T_g_*), a cold crystallization peak (*T_cc_*), a recrystallization peak (*T_rc_*), and a melting peak (*T_m_*). During the compounding experiments, the extrudate was quenched with water after mixing, and this did not give PLA sufficient time to crystallize. However, this crystallization process occurs during the DSC heating cycle above the *T_g_* and is observed as the cold crystallization peak (*T_cc_*). The formation of imperfect crystals results in subsequent recrystallization (*T_rc_*) [34]. The transition temperatures are summarized in Table 4 and remained constant even in the presence of the dyes. However, a significant change in the crystallization temperature was observed during the cooling cycle, increasing from 98.70 to 117.10 °C following the addition of alizarin (Figure 13b). Alizarin therefore appears to act as a nucleating agent, in agreement with our earlier studies [18,19].

### 3.4. Characterization of the Fiber Webs

#### 3.4.1. Effect of Dyes on the Viscosity of PLA

**Figure 14:**
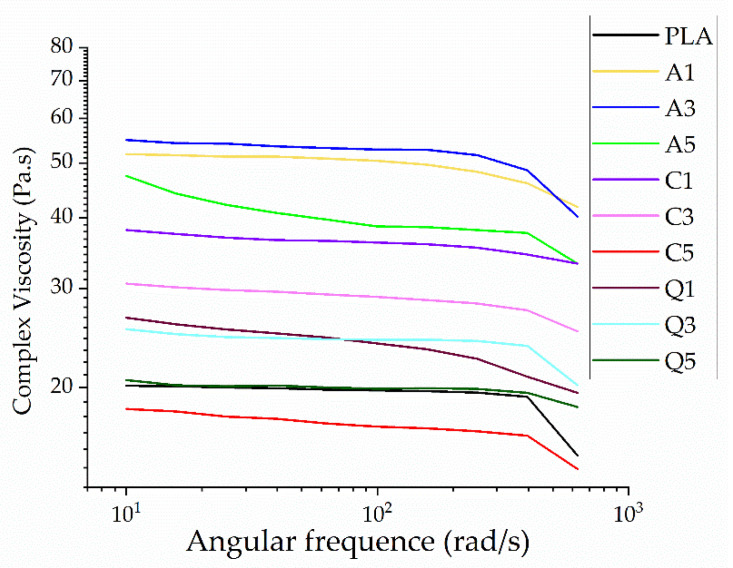
Rheogram of melt-electrospun fibers formed of PLA and its composites containing 1%, 3%, or 5% (*w*/*w*) of alizarin (A1, A3, A5), curcumin (C1, C3, C5), or quercetin (Q1, Q3, Q5).

The rheogram of PLA and its composites in the fiber web showed evidence of shear thinning behavior, where the complex viscosity falls with increasing angular frequency (Figure 14. The increase in shear force breaks the chain interactions and entanglements, causing the polymer chains to align in the direction of the applied force and making it easier for the polymer melt to flow.

**Figure 15:**
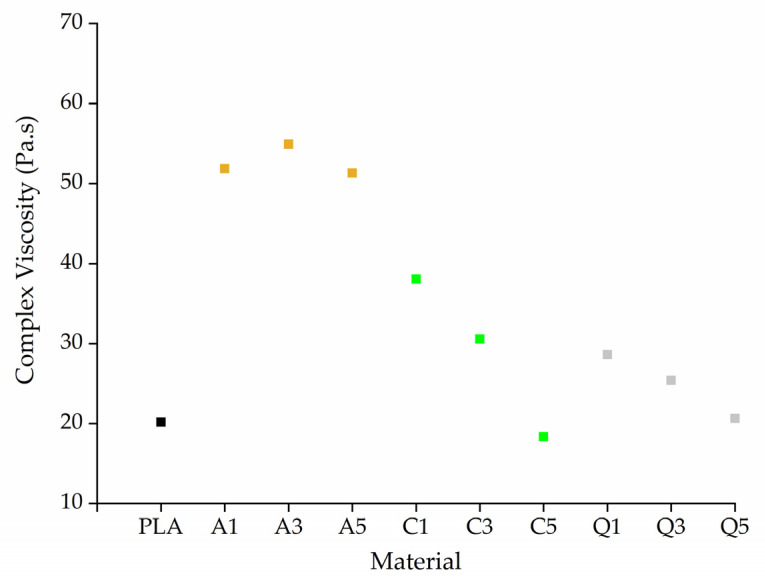
Complex viscosity of melt-electrospun fibers formed of PLA and its composites containing 1%, 3%, or 5% (*w*/*w*) of alizarin (A1, A3, A5), curcumin (C1, C3, C5), or quercetin (Q1, Q3, Q5) at an angular frequency of 10 rad/s.

For ease of comparison, the complex viscosities of all fibers are shown at an angular frequency of 10 rad/s (Figure 15). The complex viscosity increases (almost twofold) following the addition of alizarin, reflecting its existence as solid particles in the composite and the possibility of hydrogen bonding with PLA, hindering the mobility of PLA chains. The forces at low shear rates are not strong enough to break these bonds, resulting in a high complex viscosity, but higher shear causes these interactions to break. Quercetin caused a marginal increase in the viscosity of PLA but not as significant as alizarin, in agreement with our earlier results [19]. The addition of curcumin initially led to an increase in viscosity but this effect was reversed at higher curcumin concentrations. Curcumin may therefore act as a plasticizer at higher concentrations, or it may promote the degradation of PLA, which we tested by GPC analysis (Section 3.4.5). Although the addition of dyes increased the viscosity of the melt, the opposite of the effect that we sought, it only increased by 50 Pa.s. Therefore, if the dyes reduce the electrical resistance of the melt, this could still promote the formation of narrower fibers.

#### 3.4.2. Effect of Dyes on the Electrical Resistance of PLA and Composite Fibers

**Figure 16:**
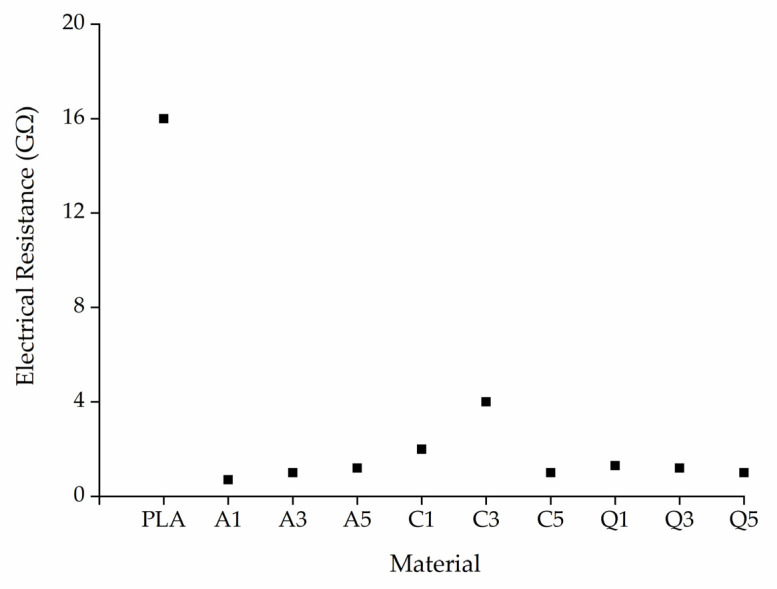
Electrical resistance of PLA and its composites containing 1%, 3%, or 5% (*w*/*w*) of alizarin (A1, A3, A5), curcumin (C1, C3, C5), or quercetin (Q1, Q3, Q5).

The electrical resistance of PLA was much higher than that of its composites (Figure 16). Even the presence of 1% alizarin or quercetin reduced the resistance to below 1 GΩ as compared to PLA, which has an electrical resistance of approximately 16 GΩ. The functional groups and double bonds present in the aromatic structures of the dyes may increase the electrical conductivity of the composites, thus favoring the formation of narrower fibers.

#### 3.4.3. The Diameter of PLA and Composite Fibers

**Figure 17:**
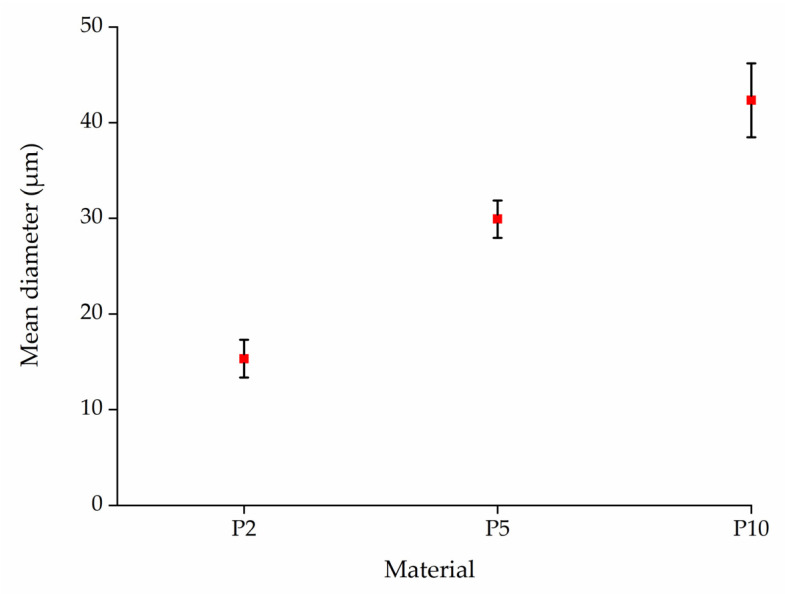
Mean diameter of fibers in webs prepared from pure PLA at different pump speeds of 2 rpm (P2), 5 rpm (P5), and 10 rpm (P10).

We assessed the diameters of PLA fibers in webs prepared by melt electrospinning at pump speeds of 2, 5, and 10 rpm (Figure 17). The mean fiber diameter increased at the higher pump speeds due to the higher volume of polymer melt passing through the spinneret in a shorter time, providing less opportunity for the polymer to interact with the electric field. The resulting coarseness of the fibers is in agreement with our earlier results [10].

**Figure 18:**
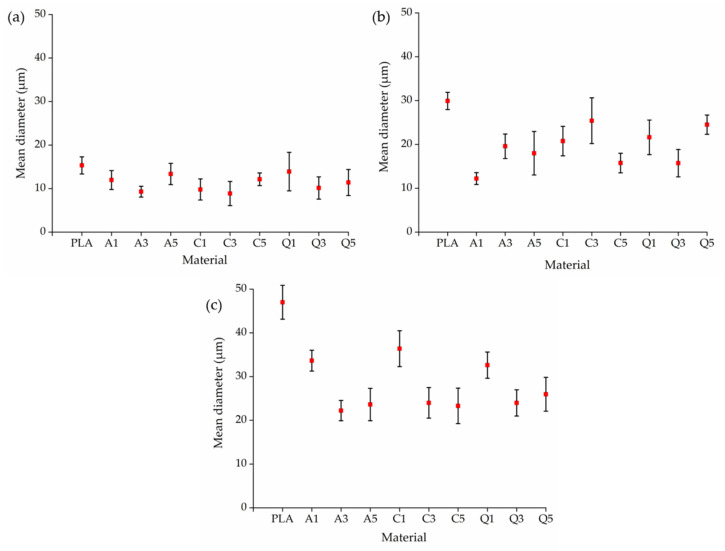
Mean diameters of fibers prepared from pure PLA and composites containing 1%, 3%, or 5% (*w*/*w*) of alizarin (A1, A3, A5), curcumin (C1, C3, C5), or quercetin (Q1, Q3, Q5) at pump speeds of (**a**) 2 rpm, (**b**) 5 rpm, and (**c**) 10 rpm.

Almost all fibers containing dyes were finer than pure PLA fibers prepared at the same pump speed (Figure 18). The highest mean fiber diameter at 2 rpm was 15 µm (pure PLA) and the lowest was 9 µm for composite C3 containing 3% (*w*/*w*) curcumin, which was also the lowest diameter that we achieved across all tests. The difference in diameter between pure PLA and its composites became more exaggerated at higher pump speeds. The highest mean fiber diameter at 5 rpm was 30 µm (pure PLA) and the lowest was 12 µm for composite A1 containing 1% alizarin, whereas the highest mean fiber diameter at 10 rpm was 46 µm (pure PLA) and the lowest was 22 µm for composite A3 containing 3% alizarin. At higher throughputs, the polymer jet flows quicker, giving it a smaller amount of time to interact with the electric field. Furthermore, the jet is also thicker and the surface area that interacts with the electric field is more limited, which inhibits the whipping of the PLA polymer jet. However, the addition of dyes led to an increase in electrical conductivity, which should counteract this effect and promote whipping, ultimately reducing the fiber diameter. As with alizarin, curcumin and quercetin also reduced the diameter of the PLA fibers. These results suggest that the increase in melt viscosity caused by the dyes is not significant enough to affect the fiber diameter and the decrease in electrical conductivity plays a greater role. It was also observed that although the diameter of the fibers was reduced in most cases upon the addition of dyes, the standard deviation seemed to have increased. In the melt-electrospinning equipment, we have a single-screw extruder followed by a large spin plate through which the fibers are made, and there is no mixing happening in the spin plate. We hypothesize that the agglomeration of the dyes can take place in the spin plate during spinning and this could lead to more non-uniformity in the fiber, therefore leading to a higher standard deviation. However, this needs to be investigated and optimized further.

We observed a similar reduction in the diameter of melt-electrospun PLA fibers when we added alizarin in a laboratory-scale process [10,19]. However, the dwell time was higher in the pilot-scale process, which caused slight polymer degradation that was not observed at the smaller scale. The fiber diameter is affected by dyes to a greater degree at higher throughputs. Since the throughputs used in the pilot scale and the lab scale are different from each other, the extent to which the dyes affect the diameter is also different. Furthermore, the nozzle-to-collector distance in the laboratory-scale process is greater (10 cm) than in the pilot-scale device (7 cm). Our results show that melt electrospinning is scalable but each parameter affecting the properties of the fiber must be optimized.

**Figure 19:**
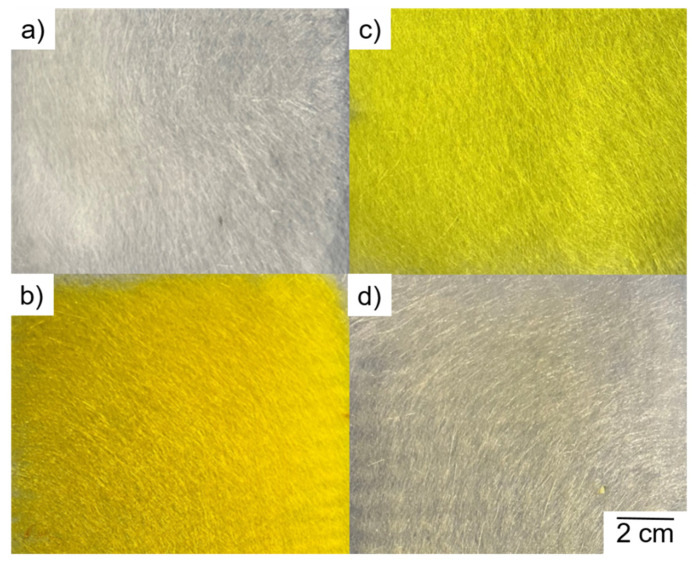
Visual appearance of melt-electrospun fiber webs prepared from (**a**) pure PLA and composites containing 5% (*w*/*w*) of (**b**) alizarin, (**c**) curcumin, and (**d**) quercetin.

We observed the uniform deposition of fibers regardless of the composition, but alizarin and curcumin achieved a higher dyeing efficiency than quercetin (Figure 19). The quercetin powder has a light yellow color but the electrospun webs were much paler, suggesting that quercetin has lower dyeing power.

#### 3.4.4. Analysis of Melt-Electrospun Webs by DSC

**Figure 20:**
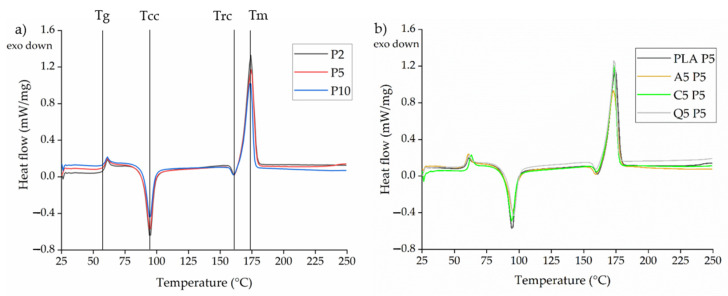
DSC thermograms of (**a**) PLA webs spun at different pump speeds (P2, P5, and P10 = 2, 5, and 10 rpm, respectively) and (**b**) PLA and PLA composite webs spun at a pump speed of 5 rpm (A5, C5, and Q5 = composites containing 5% (*w*/*w*) alizarin, curcumin, and quercetin, respectively).

The DSC thermogram of the electrospun webs revealed a glass transition at ~60 °C, cold crystallization at ~95 °C, and a *T_m_* of ~173 °C (Figure 20). The values are summarized in Table 5. In a typical melt-spinning process, mechanical drawing of the fiber reduces the fiber diameter while aligning the polymer chains, increasing the crystallinity. During melt electrospinning, the electric field is used to draw the fibers and therefore we expected a similar phenomenon. The fiber diameter was much lower at a pump speed of 2 rpm compared to 10 rpm, so we also expected higher crystallinity at the lower speed. However, the change in fiber diameter did not appear to affect the crystallinity of the PLA webs, with similar values of ~11% in each case. The crystallinity values of all fiber webs prepared from PLA composites were also similar, suggesting that varying the process parameters can reduce the fiber diameter, but the stress induced here was not sufficient to induce crystallization. Our crystallinity values were also similar to a partially oriented yarn prepared by melt spinning [35,36]. Our results show that pilot-scale electrospinning requires further optimization to achieve well-oriented fibers.

#### 3.4.5. Analysis of Melt-Electrospun Fiber Webs by GPC

**Figure 21:**
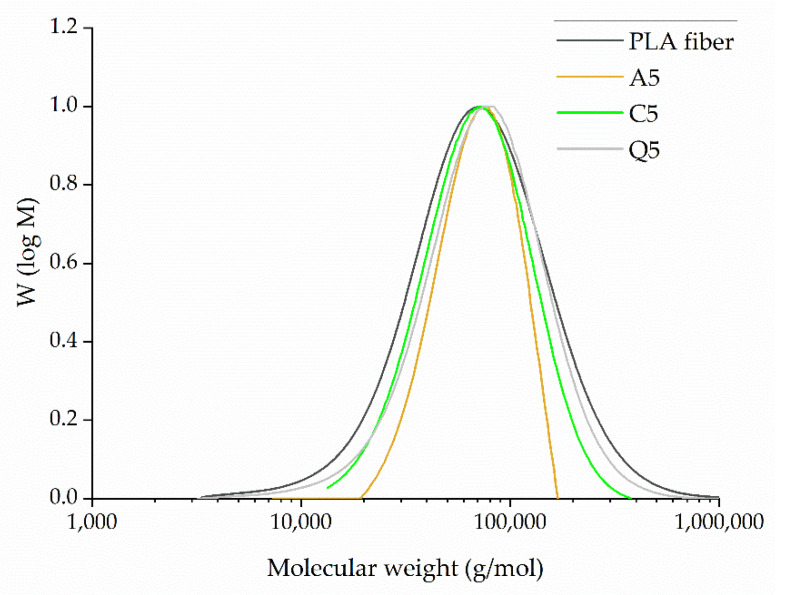
GPC elugram of melt-electrospun fiber webs formed of pure PLA or PLA composites containing 5% (*w*/*w*) of alizarin (A5), curcumin (C5), or quercetin (Q5).

The GPC elugram of melt-electrospun fiber webs containing 5% (*w*/*w*) of each dye revealed similar molecular weights (Figure 21). The average molecular weight and PDI values are summarized in Table 6. The PLA and Q5 fiber webs showed little evidence of degradation during processing, matching the values of the composites after compounding (Table 3). However, the average molecular weights of the A5 and C5 fiber webs fell below those of the A5 and C5 composites, suggesting some degradation during processing (Table 3). Even so, the amount of degradation was not sufficient to hinder the electrospinning process, allowing the continuous formation of fibers. We observed weight loss for composites containing alizarin and curcumin during the isothermal TGA step, which may reflect the release of bound water from the dyes. This may also explain the degradation of PLA during melt electrospinning. GPC analysis showed that the reduction in viscosity noted for the curcumin composites might reflect the degradation of PLA rather than the ability of curcumin to act as a plasticizer.

#### 3.4.6. Antibacterial Properties of Melt-Electrospun Fiber Webs

**Figure 22:**
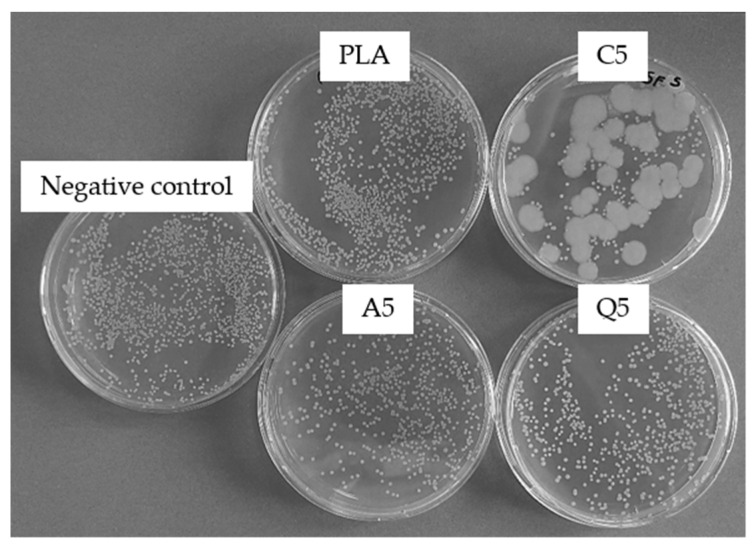
Antibacterial activity of melt-electrospun webs of pure PLA or PLA composites containing 5% (*w*/*w*) of alizarin (A5), curcumin (C5), or quercetin (Q5) against *S. aureus* at a 10^−5^ dilution.

The antibacterial properties of the PLA and composite fiber webs were tested against *S. aureus* to determine whether the properties of the dyes were retained (Figure 22). The A5 and Q5 fiber webs showed little antibacterial activity, whereas the C5 fiber web retained some antibacterial properties but was not as potent as the pure dye. Furthermore, the propagation of a potentially fungal contamination was observed using the C5 fiber web that might originate from material processing and handling. For the assessment of the antibacterial potential of the C5 fiber web, these fungal colonies were neglected. This reduction in the antibacterial activity is likely to reflect the distribution of the dye in the polymer, with only those molecules presented on the fiber surface being able to kill the bacteria [24]. Similarly, quercetin-loaded PLA fibers were previously shown to lack the leaching of quercetin while retaining antibacterial activity [26]. Since there is no leaching, there is no possibility of the dye molecule stuck inside the fiber to provide any activity. Alizarin and curcumin also lost some of their antibacterial activity after thermal treatment, which would influence the final properties of the resulting fibers. A higher concentration of dye therefore appears necessary to ensure that the antibacterial activity is retained in melt-electrospun fibers.

## 4. Conclusions

We investigated the effects of three dyes (alizarin, curcumin, and quercetin) on the pilot-scale melt electrospinning of PLA. We first analyzed the thermal stability of pure PLA and pure dyes at processing temperatures over a prolonged duration. We then tested the antibacterial activity of the dyes against *E. coli* and *S. aureus*. The dyes were effective against *S. aureus* at concentrations as low as 5 mg/mL, but the activity of alizarin and curcumin was reduced by thermal treatment and the concentration had to be increased to more than 15 mg/mL to achieve the same effect. This may reflect either keto-enol tautomerization or a change in the crystal structure of alizarin and curcumin during thermal treatment. After compounding, we analyzed the thermal stability of the PLA composites. FTIR spectroscopy revealed the possibility of hydrogen bonding between PLA and the dyes. Based on these results, we selected a melt-electrospinning process temperature of 230 °C and produced electrospun fiber webs with diameters in the lower micrometer range. The finest pure PLA fibers that we produced were 15.3 µm in diameter, but this was reduced to 9.3 µm by adding 3% (*w*/*w*) curcumin, the lowest diameter that we achieved overall. Increasing the throughput led to an increase in diameter, but all three dyes reduced the fiber diameter at all three concentrations tested. At higher throughputs, the addition of dyes led to a greater reduction in diameter. DSC analysis of the melt-electrospun fiber webs revealed that neither the additives nor the process parameters affected the (generally low) crystallinity of the fibers. GPC analysis indicated the slight degradation of PLA fiber webs containing alizarin or curcumin. Antibacterial analysis showed that only the fiber web containing 5% (*w*/*w*) curcumin retained some antibacterial activity against *S. aureus*. This is likely to reflect the small proportion of dye molecules available on the fiber surface compared to the larger proportion buried inside. More of the additive would therefore be necessary to achieve the antibacterial potency of the uncompounded dye. Our work provides insight into the scaling up of a melt-electrospinning process from the laboratory to pilot scale, and provides an environmentally friendly alternative to replace conventional solution electrospinning for the production of microfibers with antibacterial properties.

## Figures and Tables

**Table 1:** Chemical structures and melting points of the dyes used in this study [19,27].

Additive	Chemical Structure	Melting Point (°C)
Alizarin	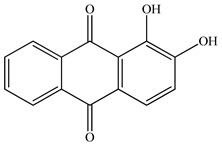	~280
Curcumin	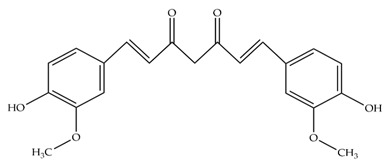	~175
Quercetin	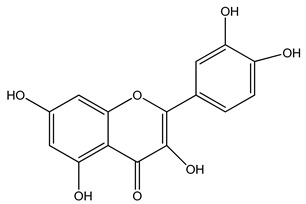	~320

**Table 2:** PLA/dye composites used for the spinning trials.

Composite Abbreviation	Colorant Name	Percentage (*w*/*w*) of Colorant
PLA	–	-
A1	Alizarin	1
A3		3
A5		5
C1	Curcumin	1
C3		3
C5		5
Q1	Quercetin	1
Q3		3
Q5		5

**Table 3:** Average molecular weights and PDI of PLA and its composites.

Sample	Mn (g/mol)	Mw (g/mol)	PDI
Pure PLA	55,000 ± 5000	105,000 ± 10,500	1.89 ± 0.3
Alizarin A5	54,400 ± 5340	105,000 ± 9800	1.92 ± 0.2
Curcumin C5	53,000 ± 5200	99,000 ± 9600	1.87 ± 0.4
Quercetin Q5	58,700 ± 5570	108,000 ± 10,000	1.84 ± 0.3

**Table 4:** Thermal transition temperatures of PLA and its composites containing 5% (*w*/*w*) of alizarin (A5), curcumin (C5), or quercetin (Q5).

Material	*T_g_* [°C]	*T_cc_* [°C]	*T_rc_* [°C]	*T_m_* [°C]	*T_c_* [°C]
PLA	59.70	95.90	160.90	176.10	98.70
A5	60.10	93.30	158.80	173.60	117.10
C5	60.70	96.10	158.80	174.00	-
Q5	62.50	95.80	157.00	174.10	-

**Table 5:** *T_g_*, *T_cc_*, *T_m_*, and *X_c_* values of melt-electrospun fiber webs.

Material	*T_g_* [°C]	*T_cc_* [°C]	*Tm* [°C]	*Xc* [%]
PLA P2	59.50	94.70	174.10	12.39
PLA P5	60.00	94.70	174.50	13.47
PLA P10	59.40	94.60	173.50	10.70
A5 P5	59.60	95.10	173.00	11.17
C5 P5	62.00	94.40	173.40	11.18
Q5 P5	60.00	95.80	173.90	12.27

**Table 6:** The average molecular weight and PDI values of melt-electrospun fiber webs formed of pure PLA or PLA composites containing 5% (*w*/*w*) of alizarin (A5), curcumin (C5), or quercetin (Q5).

Sample	Mn (g/mol)	Mw (g/mol)	PDI
PLA	51,100 ± 3000	94,000 ± 9000	1.84 ± 0.2
A5	62,400 ± 5000	74,300 ± 6000	1.19 ± 0.3
C5	57,500 ± 5200	80,600 ± 7000	1.40 ± 0.4
Q5	56,900 ± 4000	91,800 ± 9000	1.61 ± 0.2

## Data Availability

The datasets used and/or analyzed during this study are available from the corresponding author on reasonable request.
